# Social Representation of Mental Health Disorders in the Italian Big Brother VIP Edition

**DOI:** 10.3390/bs14111030

**Published:** 2024-11-02

**Authors:** Rosa Scardigno, Raffaella Gambarrota, Laura Centonze

**Affiliations:** 1 Department of Educational Sciences, Psychology, Communication, University of Bari ‘Aldo Moro’, 70121 Bari, Italy; gambarrotaraffaella@hotmail.it; 2 Department of Human and Social Sciences, Universitas Mercatorum, 00186 Rome, Italy; laura.centonze@unimercatorum.it

**Keywords:** social representations, mental health, mental illness, depression, reality show, Big Brother, entertainment, discourse analysis

## Abstract

Despite the revolutionary impact of new media, television remains a socially shared reference point for media functions, e.g., information, entertainment, and hybridized genres. Through its simplified knowledge and scripts, television reduces cognitive asymmetry between experts and the public on general and specific topics, thus having a critical role in constructing social representations. This work examines two (apparently) distant realities, i.e., mental health as a fundamental aspect of public health and popular and “light” entertainment formats like reality shows. In the past, researchers investigated media representation of mental illness in general terms alongside other types of programs, e.g., coming-of-age, dramedy television series, and children’s television programs. This study examines how depression is discursively constructed and socially represented in a case that shook the Italian public opinion, i.e., a Big Brother VIP cast member with depression symptoms. The critical discourse analysis, focusing on positioning and representations about depression, enabled us to emphasize that (1) knowledge about depression is poorly defined and participants’ reactions are mostly immature and clumsy, and (2) mass media can play an essential role in creating more mindful and complete knowledge about mental health.

## 1. Introduction: Reality Shows Beyond Mere Entertainment

Reality shows represent one of the most widespread and diversified television genres in terms of production and enjoyment: They can satisfy different audiences by dealing with completely heterogeneous topics, from healthcare to beauty [1,2,3].

Despite its massive audience and financial success, reality TV remains a highly contentious and stigmatized television genre. The medium presents a host of ethical dilemmas [4] that warrant scrutiny:

(a) It has the effective ability to represent reality despite media content being “manipulated, edited and artificial” ([4] p. 735).
(b) By its very nature, reality TV embodies a media trend towards voyeurism, sensationalism, and the obsession with exposing people’s private lives [5].
(c) The consequence is that such programs confine audiences to mere users in search of leisure, diverting their attention from relevant topics. Some studies have highlighted that reality shows contribute to contemporary moral and intellectual impoverishment [1].

However, academic research has overcome the vision of the public as passive and manipulable through media content, supporting the involvement of an audience committed to critically observing the protagonists’ attitudes and behaviors and the ideas and practices implemented by producers [6]. A further issue that has dominated the ethical debate on reality shows concerns the promotion of a life approach based on individualism and capitalism [4], emphasizing material success; opportunities to improve oneself even in a cynical way; self-expression through superficial, stereotyped aspects; and adhering to societal norms. Some research has identified a correlation between women’s exposure to reality shows and perceived social pressure to be thin [7] and an increased propensity to use tanning lamps [8].

Considered the precursor of all reality shows, Big Brother (BB) debuted in 1999 as a combination of different television genres—game shows, talk shows, soap operas, and docudramas [9,10]. Drawing on the apocalyptic Orwellian metaphor, Big Brother revolves around the forced coexistence of contestants inside a house “spied on” by the public 24 h a day [2,11]. As time passes, alliances and relationships, even romantic ones, occur between two or more people [12].

A key aspect contributing to the success of this television format is its “trans-culturality” [10]. Although the program is adapted in different countries to the national television culture of reference and the expectations of different audiences [9], its rules are applied in a mostly homogeneous manner, providing for (i) long imprisonment with the absence of any multimedia technological equipment that allows contact with external reality, (ii) the exposure of the participants’ private lives, (iii) the creation of “confessionals”, and (iv) the nominations and consequent eliminations of competitors following televoting by the audience.

In the efforts to guarantee the endurance of and the retention of reality shows across several editions, thus overcoming the eventual audience reduction due to a possible “habituation” effect, reality-based TV programs can include “coup de théâtre”, unpredictable situations, and other surprising happenings, since unpredictability can contribute to creating suspense and greater involvement [13]. In addition, public/social issues, such as racism and health, can be included in the global storytelling of a specific edition. These efforts can comply with the several motivations of reality-based TV viewership, connected not only with voyeurism and curiosity but also with self-reflection, personal insights, and even empathy activation [14].

At the intersection of these orientations, the current work deepens the role of reality shows in arguing social topics and constructing representations of mental health issues, specifically related to depression, as connected to a cast member’s happenings. This overall aim has been set in line with the following considerations: (a) In its (more or less accentuated) perceived realism, the proposal of social topics in reality shows can have wide dissemination across all audiences, thus contributing to the construction of shared knowledge about unknown content but, on the other side, risking offering simplified and poor insights, and (b) mental health disorders can in part still be considered a taboo topic. Thus, it is relevant to investigate repertoires and rhetoric emerging from TV programs. To the best of our knowledge, other studies about reality-based TV have somewhat neglected to investigate how entertainment programs can contribute to constructing social representations of depression starting from authentic experiences. The case study we proposed offers an example of incorporating characters with mental health disorders into the storyline of the reality show, not just through self-disclosure practices but also through a “live” symptomatic case. The happenings “forced” all actors—host, opinionists, and cast members—to focus on and to take a position about the protagonist’s vicissitudes and, sometimes also explicitly, on the depressive disorder. The constructing the scenario this way enabled us to deepen the contribution to the social representation of mental health disorders by answering three research questions concerning (a) what the main stances and the related rhetorical–argumentative strategies emerging from live dialogues are, (b) what the main interpretive repertoires related to depression are, and (c) how the focus on these matters evolved as the program progressed.

## 2. Theoretical Background

### 2.1. Founding Theorization About Social Representations

Despite its sociological applications, the theory of social representations (SRs) acquires strong psycho-social value thanks to Serge Moscovici’s contribution [15,16,17] to define how knowledge is constructed, shared, and interpreted by individuals belonging to the same societal reality [18]. These are symbolic and specific forms of shared knowledge processing within large groups, not necessarily coinciding with the entire society. By providing models based on common sense, they transform what was initially unknown and complex into something familiar and simple. SRs are crucial in knowledge dissemination, individual and group development, and social transformation [19].

Although SR theorization includes many aspects, a particularly consolidated point concerns the description of two basic processes—anchoring and objectification, which reflect the cognitive–social soul of the construct [20]. They aim to anchor and stabilize contents (cognitive function) and create/maintain collective identities and balances (social function). Specifically, anchoring enables one to explain, name, and classify something unfamiliar by relating and integrating it into already-known social categories. This allows the well-known ones to be transformed into novel concepts. Cultural assimilation allows unknown ideas to become part of society’s collective reference frames. Anchoring is used to reduce and control fear of an unknown element. The mass media exploits this process sometimes in superficial and speculative ways; for example, social phenomena and events are linked to experiences of fear and threat or feelings of anger, pity, and compassion [21].

Objectification turns abstract concepts into images and materializes images and ideas; through this process, an object can be quickly simplified, imagined, and diagrammed. Through selective construction, the object’s different characteristics are extrapolated from the context and sorted according to cultural criteria (groups do not have equal access to information about the object) and normative criteria (only what agrees with the system is maintained as group values). Objectification is a more active and demanding process than anchoring, which occurs almost automatically whenever we face new phenomena [22]. The media uses this phenomenon when scientific concepts are transformed into images that replace original thoughts and ideas to become constitutive elements of the phenomenon.

### 2.2. Social Representations of Mental Illness

Mental illness is an unfamiliar construct represented over time. Its complexity is linked to intrinsic factors such as heterogeneity and multiple problems. People with mental illnesses often struggle with the symptoms of the illness itself, with misunderstandings and reactions from society, including various forms of stigma [23]. As a further complication, some people may apply learned and shared stereotypes concerning mental illness to themselves, creating a self-stigma paradox [24].

The body of knowledge and beliefs relating to mental disorders can promote and support positive mental health [25]. An incorrect representation of mental distress can (a) hinder understanding mental illnesses [26], (b) create discriminatory attitudes and beliefs [27,28], and (c) discourage seeking professional assistance and following medical treatment.

In the case of a complex and delicate pathology such as depression, some studies have highlighted how it is rarely recognized as a mental disorder [25,29], mainly associating the symptoms with a normal period of crisis the individual experiences throughout life and therefore not requiring medical intervention. Individuals who have been in contact with mentally ill people are more likely to recognize disorders.

More recently, Roelandt and colleagues [30] investigated the social representations of the “mentally ill” and the “depressed”: In the former case, these are images characterized by violence, danger, and fear [26,30,31], including the perception of dysfunctionality, incompetence, and irresponsibility [26,30,32]. Depression is associated with sadness, withdrawal, social isolation, and attempted suicide. Usually, it is linked to an environmental etiology (e.g., personal or professional trauma). However, this disorder is rated more positively than other mental disorders [29,32]. This could depend on the recent emphasis on the disorder’s biological and genetic determinants [26,31,33]: Depression is triggered by the interaction between traumatic environmental events and genetic predispositions [31] and is among the disorders that can benefit most from treatment [29,32,33]. Furthermore, since it is not perceived as an actual mental illness by public opinion, it is more easily tolerated by society [25,29].

### 2.3. Social Representations of Depression in Mass Media

Several studies have shown how mass media contributes to the spread and maintenance of stigma regarding mental illness [34] since the representation can be distorted through the transmission of exaggerated, inaccurate, and wrong information.

According to a recent review [35], three aspects of mental illness have been explored in English works relating to mediated communication: (1) representation of mental illness in the media (including nonfiction news media, fictional media, direct-to-consumer advertising, and social media), (2) impact on the public, and (3) opportunity to reduce stigma through the media. Depression emerged as the core of the article in 12 out of 41 cases. This was especially relevant to DTC and social media. Media portrayals of mental illness are dangerous, connected to criminals, and violent.

Depression is described as a chemical imbalance [36]. This image could significantly impact help- and treatment-seeking behavior [36]: The emphasis placed on the biological and social causes of depression could explain the widespread belief that it is more easily treatable than other disorders using psychotropic drugs [33]. Furthermore, depression elicits more positive emotional reactions than schizophrenia [37]; it is less associated with dangerousness and violence [37].

Despite these findings, there are various stereotypes frequently associated with people with mental health conditions such as depression, including lack of control, violence, incompetence, weakness, and suicidal tendencies [26]. After examining several posts published on the Sina Weibo platform, Wang and Liu found multiple devaluing comments towards depressed subjects. They also found derisive attitudes and a refusal to recognize depression as a critical illness. Similarly, a German study demonstrated how news of a plane crash caused by a depressed man reinforced the belief that mental illness and depression are associated with unpredictability [38]. In young people, depression is often associated with suicide, alcohol abuse, and bullying [39]. Various media contents thus emphasize the need to protect depressed people from self-destructive tendencies [34].

Psychological literature [40] suggests that mood disorders, and in particular, depressive disorders, are more convincingly depicted on television than on other outlets. Mentally ill people are typically labeled as “crazy”, “confused”, “difficult”, or “wrong” by the media [41]. Most mental disorder sufferers were mocked and removed from the workplace, and 50% were forced to hide their disorder.

## 3. The Research

### Procedure, Materials, and Methods

During Big Brother VIP’s seventh Italian edition (September 2022–April 2023), events in the first month led to an absolute media case. After only 10 days in the Big Brother house, Marco Bellavia (former television presenter and actor) decided to leave the house and withdraw from the game, following oppressive and denigrating attitudes by some of his housemates towards behaviors that were later traced back to depressive symptoms. Following Bellavia’s exit from the house, Big Brother VIP took measures against those with highly negative attitudes. The episode during which the topic was discussed for the first time reported a 25.1% television share, with 3,313,000 viewers. This percentage is far higher than the first (21.73%) and the last episodes (24%), which are usually the most followed. This case shocked the Italian audience and public opinion, even entering the media and public agendas.

This work investigates how depression and mental illness have been addressed in the most famous Italian reality shows, thus contributing to their social representation construction/consolidation. The focus is not only on what has been said but also on how it has been said, referring to communication processes in the media context, positionings, and emerging sense-making dynamics. The analyses aim to detect how, starting from a depressive episode experienced on TV, mental health and depression became topics of conversation and oriented public attitudes and decisions during the Italian BB. Specifically, our research questions aim to gain insights into:

(a) The dialectics of positioning concerning the stances taken in topic discussion and the related rhetorical–argumentative strategies;
(b) Depression-related content and interpretive repertoires;
(c) The presence and evolution of the theme within the Big Brother VIP format.

The dataset was extrapolated directly from Big Brother VIP’s seventh edition episode archive on Mediaset Infinity (https://mediasetinfinity.mediaset.it/) (accessed on 1 September 2024). After watching all the episodes broadcasted on TV, we found that the topic of mental health disorders was discussed in four episodes (3, 6, 10, and 20 October 2022). In the first one, even though the protagonist was not in the TV studio, the TV share was 25.1%, gathering 3,313,000 persons as the audience. This is higher than the share concerning the first (21.73%) and the last (24%) episodes, which are usually the most attended.

The involved TV episodes were transcribed and analyzed through critical discourse analysis [42] in terms of markers of diatextual analysis [43]. This type of discourse analysis (DA) procedure takes shape in the “SAM model”, with various markers outlining the three main components of meaning construction between text and enunciation context:

- Subjectivity refers to the image the interlocutor builds of himself and his ideal interlocutor through markers of agency, affectivity, and débrayage or embrayage, which modulate detachment or involvement, agency or inaction, closeness, or distance when talking about a specific issue;
- Argumentativeness, concerning how discourse meanings and objectives emerge and are organized through enjeu markers, narrativity markers, and the logoi–antilogoi network;
- Modality, concerning discourse manner and form, through the metadiscursive genre, the discursive genre, and opacity markers such as metaphors and other rhetoric figures.

Diatextual analysis is a hermeneutic procedure by which the analyst can integrate a bottom-up and a top-down pathway [43]. Specifically, while searching from SAM markers and features through a paper-and-pencil procedure, the core aim of this kind of DA is to comprehend better how the “context” is seen by the “text’s enunciators”, thus implying the need for revealing the hidden “enjeu” that lays inside any communicative effort. The diatextual analyst works in line with the following steps: Firstly, s/he sets some more or less specific research questions concerning discourses, considered meaningful social events. Second, s/he selects a corpus and, if necessary, s/he transcribes it. This allows him/her to carefully read it multiple times. Third, based on his/her knowledge about the subjectivity, argumentativeness, and modality markers, s/he tries to identify eventual occurrences and co-occurrences that can represent some regularities. Fourth, by matching bottom-up and top-down pathways, s/he proposes assumptions that interpret the emerging regularities in line with the context, meant as the whole text, the discursive genre, the sociocultural scenario, and its expectations [43,44].

For synthetic reasons, the main argumentative and rhetoric dynamics will be outlined; however, excerpts and examples of the above-exposed markers will be proposed. The next section will outline the positions taken by different actors and interpretative repertoires concerning depression and mental illness [44].

## 4. Results

### 4.1. Actors, Positioning, and Stances

Using conversations from the reality show, we will analyze how different “actors” position themselves and how depression is represented in the dialogue.

In the Italian edition of BB VIP, the host plays a crucial role in managing the different moments of this format, acting as a figure who, through extensive use of metadiscourse, orients the viewers’ understanding and favors the most suitable interpretations. In the case study, this rhetorical function is exercised along three fundamental axes:

(a) The narration of events is organized through the proposed synthesis, the memory of events, the anticipation of some elements, and the attestation that the narration will continue later. These proposals emphasize the discussion and create suspense, discouraging the audience from changing the channel.
(b) The emotional component is emphasized through the massive use of affective markers to create emotional closeness, intimacy, and involvement.
(c) The host demonstrates empathy for mental health issues, commitment, and emotional participation in the protagonist’s pain. This strategy is intended to make the audience feel the host’s genuine concern and involvement in the show’s themes.

Extract 1: “And this story involves all of us a bit. Why? Because this program is about life and life is also made up of this. It is made of lightness, but it is made of pain. And Marco’s pain is everyone’s pain, because how many times have we found ourselves without someone to lend us a hand, without someone to tell us how you are, who sees us dying in a corner and says nothing? Unfortunately, this also happens, Big Brother tells it in its rawness, in his truth. Could he make this all sick? Maybe, it’s a punch in the stomach, for sure”.

Extract 1 exemplifies three orientations: The framing of the case is rendered through the emphasis on the reality, albeit grim, of the format as well as on its generalization (“us”). A recurring theme in the entire dialogue, i.e., the lack of help, is immediately proposed, using the metaphor that best symbolizes it: The “hand” expected by those in difficulty is “not given”. This metaphor emphasizes the emotional impact, implying the intention to make the audience feel the pain. Then it closes again, symbolizing the painful and debilitating “punch in the stomach”.

The reference to the need to resort to “punishment” after what happened plays an equally important role in reconstructing the story.

Extract 2: “As you can imagine, we reflected and analyzed hours and hours of footage. We are not here… it is not a trial, but unfortunately, over the last few days, some observations have been aired that cannot be accepted, which have aroused the indignation of many Italians, of many people who follow this program with affection”.

The play between “you”, and “us”, and generalizing expressions regarding the public (“many Italians”), involves the public by making it both part of the decision-making process and protected as a target “offended” by events. This necessity is, however, accompanied by disclaimers (“It is not a trial, however…”) aimed at mitigating the extent of the incident and anticipating the educational scope of the measures not yet adopted.

However, the host’s position oscillates between rejection and acceptance, justification and condemnation, and anger and sympathy towards its protagonists.

Extract 3: “I swear to you, you don’t know how angry you are making me right now because you are a truly special girl; you are a great competitor in this edition, but you said something inconceivable. But how do you tell someone ‘You deserve to be bullied’? No way! […] It’s a complicated evening, you know; these images of Ginevra are heartbreaking for me, and I think for everyone because she is truly a good girl, but she said something unforgivable”.

This ambivalence and the declaration of its complex management (“I swear to you”), strengthened through various verbal expressions (“It’s a complicated evening”) and extended to the public (“I think for everyone”) culminates with the metaphor of “heartache”, generally used in situations of evident closeness and emotional difficulty.

Besides the host, the Italian “opinionists” are crucial in critical moments. In the case study, the rhetorical function of the interventions proposed by the two women seems functional:

(a) To confirm dualism, intertwining arguments of distance and familiarity, rejection and acceptance, and empathy for the victim, and casting doubt on members’ understanding;
(b) to normalize depression, defined as “our society’s disease”.

In general, the discursive choices and argumentative elements combine to show, on the one hand, the denunciation of the greatest responsibility attributed to the competitors in the house, namely, indifference to others’ pain and, on the other, attestations of understanding and attempts at rapprochement to avoid summary condemnation.

Looking at the cast members’ positioning, three main stances were found:

(a) Those who immediately apologized to Marco.

Extract 4: “I regret it, I’m sorry, I deeply feel guilty. I take back all my words; that’s not why I’m here. The day we were informed that Marco had left, I felt that there was something serious. […] I can only be ashamed, I don’t know how… it was an unfortunate phrase, and I don’t want to justify those behaviors; I repeat, I suffered from them as a teenager, and that’s why I’m ashamed of it”.

Apologies are accompanied by repentance, sorrow, and awareness in an ascending climax that makes guilt explicit. The metaphor for the “bath of shame” is particularly effective at indicating the pervasiveness of this feeling. Consequently, the competitor experiencing elimination conveys the disclaimer, “That’s not the reason I am here”.

(b) Those who justify themselves by limiting responsibility for their actions or failures. This position is articulated through the use of the most common forms of “moral disengagement” [45]: (i) distancing/diffusion of responsibility using impersonal formulas and first-person plural (“I didn’t understand his discomfort, we couldn’t understand what was happening at all when we found him on the ground we couldn’t tell if he was crying or praying”), (ii) reference to higher-order principles and legitimization of one’s viewpoint by reference to professional authority (“He sought a professional opinion which I gave him, and you can’t lie about something that’s wrong, you can’t say ‘but you look great come on’. If someone is not well, the other must say ‘look, you’re not well”), and (iii) euphemistic labeling (“So it certainly wasn’t a good joke, but it was meant to be a joke”) and shifting from moral to aesthetic evaluation (“I think that the sentences that have been said are in very poor and bad taste, but not in bad faith”), (iv) attribution of blame to the victim (“… because Marco had an offensive attitude towards me and invaded a private and physical space without consent and upset me”). These stances are articulated through the adversative conjunctions “but” and “however” and the argumentative “because”, which seek to justify one’s actions and converge in an explicit reduction of responsibility (“But I don’t feel guilty about him, I feel like saying this”).
(c) House members who have shown emotional closeness.

Extract 5: “I think I’m as special to him as he is to me. In fact, I told Gegia she had made a mistake. This hurt me a lot, even seeing those images of him on the ground. Those images devastated me, like, something that really punches you in the gut that you can’t breathe. I can’t wait to see him, hug him, and talk to him”.

The closeness towards the protagonist is rendered through reciprocity, with a strong emotional expression of pain towards his condition. Metaphors elicit the contrast between the different forces hidden in the “fist”, in the embrace and the will to continue outside the context of the reality show, where the relationship started. Among those embracing the latter position, regret also emerges as linked to the feeling of being able to do more.

### 4.2. Representing Depression

When depression is at the core of the discussion, the host, commentators, and cast members show ambivalence in their positioning. Narrations, interventions, and requests for explanation counterbalance uncertainty in defining and treating the issue.

Extract 6: “Marco Bellavia has expressed an uneasiness, a psychological fragility inside the Big Brother house, which has become increasingly worse in the last few days, immediately after our episode, which caught us completely off guard. That shocked us and filled the entire audience with an emotional current, which expressed itself in a supportive and united way towards his suffering and discomfort. And who teamed up with him; he was increasingly alone against a group, the others in the house. It’s a bad story, I feel like saying, it’s a story that has neither winners nor losers, on the contrary. It has many losers”.

During episodes, the host mostly defines depression as “discomfort”, “fragility”, and “suffering”. Depression is inserted into a story that presents the reactions—of disorientation but also emotional involvement, the condition of the protagonist—of solitude but also solidarity, and morality—defined by many losers. Also included are faults and responsibilities related to indifference, selfishness, and lack of empathy.

Extract 7: “This topic, that of emotional fragility, call it what you want, depression, is extremely important and widespread. In our country, we never talk about depression, the loneliness linked to it, the sense of shame you feel in being depressed, panic attacks, not being able to communicate with the rest of the world, pulling down all the shutters to close contact with everyone. These very widespread things are never heard on television”.

Despite the difficulty of calling this disease by its name (“Call it what you want”), the host expresses all the contrast between the relevance and diffusion of pathology and the taboo promoted by television. The difficulty in finding the right words for such a delicate topic is thus supported by rhetorical devices such as the use of the asyndeton, which gives speed to the speech, and the ascending climax, starting from depression and moving on to its experiences (solitude and shame) and behavioral consequences (lack of dialogue and closure of contacts).

Cast members talk about depression with more emotional involvement.

Extract 8: “I’ve decided that I can’t stand staying here because it creates… monsters inside. Exactly, that is, exactly like that, of anguish, of anxieties. I’m tired. Honestly, I would leave. With all due respect to you, the program, and the production. But I can’t take it anymore; I would like to go and rest for a while (*crying*)”.

Extract 8 presents the crucial moment Marco decides to leave the house, confessing his uneasiness. Despite the inevitability of the decision, this extract expresses the dignity that goes along with it.

Extract 9: “Oh yes, because in any case, I think it’s even easier for more sensitive people to get this demon, I always call it a demon because I don’t like calling it depression”.

Extract 9 is taken from another competitor, Luca Salatino, who showed emotional closeness towards Marco, talking about his past depression. He uses the metaphor “demon” to indicate this psychological distress, a word he prefers to “depression”.

These extracts contribute to the construction of two interpretative repertoires: The first concerns depression as a distressing experience, as evidenced by the words “monsters” and “demons”, and by the need to “rest”; the second concerns depression as an elusive condition, as it emerges from the difficulty of naming this disorder, but also from the reactions of others, oscillating between fear, disbelief, and inability to understand the symptoms and behave accordingly. At the intersection of these reactions is the position of those who justify themselves as inadequate to the situation. In two different moments, the protagonist seals this repertoire.

Extract 10: “I saw a person who was a bit lost in… as if I were drunk; I spent two days in the house drunk, not understanding what I was doing”.

Extract 11: “You didn’t understand but not because of you, but because I had something on my mind, which left me bewildered”.

In extract 10, Marco once again shows the difficulty in defining his condition, almost distancing himself from the self in the house (which he speaks of in the third person) and looking for an “anchoring”, which he finds in the condition of bewilderment similar to a hangover. A further passage that confirms the interpretative repertoire concerns the attempt to remove responsibility from his companions for what happened and by attributing the “blame” to himself (extract 11) through some metaphorical expressions capable of transmitting the sense of experienced vagueness, indeterminacy, and confusion (“I had something on my mind”, “bewildered”).

Further evidence of ambivalence occurs in the contrast between the “ridiculousness” of the contestants’ daily lives and the “seriousness” of the episode’s presentation of mental illness.

Extract 12: “G.: So why are you doing this? Are you crazy?

M: A little, yes, but…

G: You’re not well, you need to get treated, you’re not well”.

Extract 13: “P: If you have problems, stay at home, my son, it’s not like you come here to do psychoanalysis for free.

E: Exactly no, then go there, to the Neuro Delirium unit. (laughs)”

Extract 14: “So, for me, you deserve to be bullied, you deserve it”.

Extract 15: “D: Look, Alfonso, the only thing I’m sure of is that it takes much patience, much courage, and much willpower, and everyone must find this strength within themselves”.

Extract 16: “A: Sometimes it happens, maybe one can make it, but there are psychologists, psychiatrists, medicines that we really remember. Psychotropic drugs are not monsters; they are aids for illnesses, for syndromes like these. Among other things, today is mental distress day. It is indeed mental distress day, and I think it is very important to talk about this, as we have all been through it at least once in our lives”.

Excerpts 12, 13, and 14, replayed from the episodes as they are identified as deserving of sanctions, contain denigrating and stigmatizing vocabulary (“crazy”, “Neuro Delirium”); these verbs objectify the person (“come”, “go”) and the need for nefarious treatment (“You deserve it”). Conversely, a message of solidarity emerges at various moments, as evidenced by extracts 15 and 16, in which the focus is on personal strength (15) and external support (16), i.e., professional and pharmacological, as well as in a broader socio-cultural message that enhances the “public” nature of mental distress.

### 4.3. TV Evolution in Framing Depression

Depression treatment follows a sinusoidal trend, which alternates moments of anger towards what happened to solidarity towards Marco. The show also alternates moments of narration by the host with summary “clips”, from the implementation of “sensational” measures to the opinions of the public in the studio and at home, and from the involvement of the “executioners” to the “victim’s” rapprochement and progression.

Can episodes and mental health treatment be classified as “happy endings”? At first glance, the answer is negative, considering the statements of the three protagonists:

(a) The protagonist, when he thinks he has failed in his mission, metaphorized by the image of the battle (“M: But if I go home, I won’t carry on my mission, which for me is to make others understand that in addition to physical pain, mental pain can also be overcome”);
(b) The executioner admits her superficiality and the traps in dealing with mental disorders (“E: I too, certainly slipped into the trap of… perhaps prejudice. Perhaps now, in hindsight, I could have helped him; that is, if I could. I could have empathized and instead, I stopped at Marco’s door”);
(c) The host, who tries to take the broadcaster’s perspective in safeguarding the program and the public’s interest (“A: It is the public who said, ‘No, we don’t want this television”).

Conversely, the same positions take on more optimistic and uplifting tones during episodes. In particular:

(a) The protagonist, who reworks his own experience as a success (“And suddenly I understood, even through your discomfort, that I had succeeded in something significant: making my life experience, my weaknesses, my demons. I wanted to send a message that only together can we suffer less; only by sharing pain can we make it less heavy and unbearable”);
(b) One of the executioners, who proposes a rehabilitation of his image through the admission of guilt, requests an apology and the affective gesture of the embrace (“Then I Marco must ask you sorry, sorry for not believing you, understanding you, but above all I must apologize to you for my superficiality. I was superficial, perhaps too caught up in the game, and I didn’t look at what was beyond. I’ve been looking for you for 20 days through your agent. I hope he told you, and I would have. I want to hug you, and this is my only desire right now”).
(c) The host, who carries out a more general assessment, going beyond the specificity of the case (“I firmly continue to believe that BB is a formidable tool for addressing issues of social relevance, very important (applause), which until now had remained unheard of, in the sense of unheard, and in my opinion, this has become a beautiful piece of television”).

## 5. Discussion

Mass media plays a fundamental role in constructing collective knowledge, orienting public opinion, and shaping social representations [46]. Regarding mental illness and depression, several studies have shown that these communication tools contribute to the spread of inaccurate and distorted information, fueling the stigma linked to these problems [34]. Although depression is usually perceived more positively than other mental disorders [33], there are still many widespread stereotypes about depressed people, such as lack of control, violence, and weakness [26]. This underscores the crucial need for accurate representation in the media.

However, to the best of our knowledge, research about those matters neglected to investigate the opportunity for these sensitive contents to be socially constructed through pure entertainment formats, such as reality-based programs, with wide and differently motivated audiences. Our work aimed to fill this gap by focusing on what and how a depression case, acted out by a cast member of the Italian BB, was discussed, and the urgent societal implications of this representation.

The first research question concerned the dialectics of positioning and the related rhetorical–argumentative strategies. Within such a highly dialogic context as the reality show, where the dialectical value is repeated in the interaction between the different scenarios—inside the house, between home and studio, medium and audience—the construction of such a delicate and complex topic occurs not only through direct references but also through positioning and relational dynamics towards the competitor “carrier” of depression. In line with the roles within the group, the host organizes the narrative, orders events, and enhances the broader scope of the issues addressed in a scenario that encourages ambivalent attitudes and maintains the ranks of the program while safeguarding the dignity and relevance of the topics. The commentators in the studio confirm this ambivalence and turn it into polyphony in the house: Far from representing a compact group, the remarks, especially “catty” ones, appear different and even characterized by moral disengagement, with the clear intent of justifying their behavior and mitigating moral responsibility. In other words, on the one hand, both hosts and commentators have an ambivalent attitude toward cast members, ranging from justification to condemnation, but are engaged in the effort to normalize depression, defined as “our society’s disease”. On the other hand, competitors have a multifaceted attitude toward the protagonist’s disclosure, ranging from emotional closeness to moral disengagement, thus creating a frame of “innocence” beyond the more expected apologies. The dialectic of this positioning is constructed through a rhetorical–argumentative ambivalence, ranging from the use of emotional cues to affective markers, metaphors, and more rational arguments, especially in normalization and moral disengagement efforts.

The second research question aimed to deepen the emerging depression-related contents and interpretive repertoires. Qualitative analysis of the social construction of depression seems to confirm a general difficulty in recognizing depressive symptoms as linked to a real mental disorder [25]. A dual representation of mental health and depression has emerged. On the one hand, there is full awareness of the need to publicly deal with these issues to raise public awareness of the widespread discomfort. On the other, the issue is not appropriately addressed: The interpretative repertoires of “distressing experience” and “elusive condition” create uncertainty, unpreparedness, and embarrassment in coping with the pathology. The protagonist uses anchors (“as if I were drunk”) and attempts to reduce others’ responsibility (“It’s my fault”), which, on the one hand, mitigates the issues implicitly linked to shame and stigmatization, but on the other reinforces stereotypical views and self-stigma processes [24]. The objectification is more complex and emotionally oriented: In line with the theorization defining images as replacing the original thoughts [22] and becoming constitutive elements of the phenomenon itself, the metaphors of monsters and demons risk consolidating a disturbing and challenging scenario about the disease. No references to danger, violence, or fear emerge, probably for either the character (also known to the Italian public for his commitment to children’s programs) or for the frame—the reasonably controlled and protected situation of a reality show—which can orient the image transmitted to the public differently [47]. Whereas interpretative repertoires, which are mainly constructed by all the actors involved in BB, confirm a wider difficulty in talking about mental health disorders, the anchor/objectivation dynamics, mainly acted out by the protagonist, testify to the efforts to make depression more concrete and “relatable”, even in the public sphere, thus empowering the personal witness as more effective.

The third research question investigated the presence and evolution of the theme within the Big Brother VIP format. Both in the replayed episodes and in ex-post reconstructions, participants exhibit more insecurity and superficiality in their behaviors than concern since they are aware of being controlled. Consequently, the denigration [41] and derision [26] towards the protagonist appear paradoxical, entailing the need for Big Brother’s intervention. Furthermore, the host initially depicts the general tension using the metaphor of a story without winners and losers. However, throughout time, more explicit attempts to demonstrate emotional closeness towards people suffering from depression are found; personal experiences and vicissitudes are shared, which contribute to “normalizing” depression; and even the rehabilitating reflections concerning the “executioners” and requalifying concerning the educational scope of the “reality” program arise as attempts to demonstrate a “happy ending”, with people being punished but having “learned the lesson”.

The answers to the three research questions proposed through the diatextual analysis of the discourses from the four episodes of the Italian seventh edition of BB VIP devoted to these issues converge in the individuation of complex and multifaceted social representations about mental health disease. On the one hand, it is evidently a “hard” topic to manage. This overall difficulty, which can be related to the unfamiliar topic as well as to its taboo nature, is quite noticeable in disarranged attitudes and behaviors, ambivalent positioning, multifaceted argumentation, and evasive repertoires. However, the answer to the third research question can add a new light on these matters: It seems that the strive to talk about mental health disorders in a more organized and mindful way supports the construction of more positive representations, where the critical issues are still open but can be approached more confidently.

## 6. Conclusions

Similar to some cases of TV series, entertainment media incorporate characters with mental health disorders into their storylines [48] as part of the global effort to contribute to normalizing mental illness and neurodiversity [49]. The proposed case study from the Italian BB is interesting since it focuses on a character belonging to “very important persons” and a more general glazed world that often stigmatizes any potential weakness [50].

This work has some strength points, specifically related to (a) the attention set on the different discursive ways of talking about depression in BB, founded on lexical choices, stances, and positioning; (b) the emphasis on the several personal attitudes towards depression and mental illness in the general frame of the reality show; and (c) the determination to follow the evolving happenings, both on the part of the lay participants and on the part of the presenter, thus emphasizing framing practices and gradual, slow shifts. These points conjointly contribute to emphasizing the importance of the topic and to improving some global reflections about mental illness and depression, and their representations in public/popular opinion.

Even though several advances have been made in the media regarding mental illness and depression, eliminating the stigma remains a challenge. The reflections set out here represent an important starting point towards the identification of strategies and methodologies for the media’s representation of depression, which can raise public awareness and provide a truthful image of this psychiatric condition, definitively overcoming the impression that it is a taboo topic, supporting the development of destigmatization strategies [30], and combating the phenomena of (self-)produced social isolation.

### Limitations

This work presents some weaknesses. One of the main limitations concerns the impossibility of defining what effects the media’s management of a depression case whose symptoms became apparent “live” in the context of a reality show notoriously aimed at entertainment may have had on the representation of the public, having investigated the case from the “broadcaster” viewpoint. Nonetheless, to our knowledge, this is the first attempt to consider the theme of mental health and depression in the context of a reality show, hoping that the various “contamination” forms that the program has experienced over the years can contribute to an educational scope, analogously to what has already been carried out in some cases with edutainment programs [51], and maybe also reduce the stigma and self-stigma linked to mental illness.

## Data Availability

The datasets presented in this article are not readily available because they are part of an ongoing study involving cross-cultural analysis and parallel corpora. Requests to access the datasets should be directed to corresponding author Dr. Rosa Scardigno (rosa.scardigno@uniba.it).
